# MicroRNA-1296 inhibits metastasis and epithelial-mesenchymal transition of hepatocellular carcinoma by targeting SRPK1-mediated PI3K/AKT pathway

**DOI:** 10.1186/s12943-017-0675-y

**Published:** 2017-06-12

**Authors:** Qiuran Xu, Xin Liu, Zhikui Liu, Zhenyu Zhou, Yufeng Wang, Jianfeng Tu, Lijie Li, Hangxing Bao, Liu Yang, Kangsheng Tu

**Affiliations:** 1 Key Laboratory of Tumor Molecular Diagnosis and Individualized Medicine of Zhejiang Province, Zhejiang Provincial People’s Hospital (People’s Hospital of Hangzhou Medical College), Hangzhou, Zhejiang Province 310014 China; 2 Department of Neurosurgery, Zhejiang Provincial People’s Hospital (People’s Hospital of Hangzhou Medical College), Hangzhou, Zhejiang Province 310014 China; 3 Department of Hepatobiliary Surgery, The First Affiliated Hospital of Xi’an Jiaotong University, Xi’an, Shaanxi Province 710061 China; 4 Department of Hepatobiliary Surgery, Sun Yat-Sen Memorial Hospital, Sun Yat-Sen University, Guangzhou, Guangdong Province 510120 China; 5 Department of Emergency, Zhejiang Provincial People’s Hospital (People’s Hospital of Hangzhou Medical College), Hangzhou, Zhejiang Province 310014 China; 6 Department of Gynecology, Zhejiang Provincial People’s Hospital (People’s Hospital of Hangzhou Medical College), Hangzhou, Zhejiang Province 310014 China; 7 Zhejiang Hospital of Traditional Chinese Medical, Hangzhou, Zhejiang Province 310006 China

**Keywords:** MicroRNA-1296, Hepatocellular carcinoma, SRPK1, Metastasis, PI3K/AKT pathway

## Abstract

**Background:**

Increasing evidences demonstrate that miRNAs contribute to development and progression of hepatocellular carcinoma (HCC). Underexpression of miR-1296 is recently reported to promote growth and metastasis of human cancers. However, the expression and role of miR-1296 in HCC remain unknown.

**Methods:**

The levels of miR-1296 in HCC tissues and cells were detected by qRT-PCR. Immunoblotting and immunofluorescence were used for detection of epithelial-to-mesenchymal transition (EMT) progression in HCC cells. Transwell assays were performed to determine migration and invasion of HCC cells. A lung metastasis mouse model was used to evaluated metastasis of HCC in vivo. The putative targets of miR-1296 were disclosed by public databases and a dual-luciferase reporter assay.

**Results:**

We found that the expression of miR-1296 was reduced in HCC tissues and cell lines, and it was associated with metastasis and recurrence of HCC. Notably, miR-1296 overexpression inhibited migration, invasion and EMT progress of HCCLM3 cells, while miR-1296 loss facilitated these biological behaviors of Hep3B cells in vitro and in vivo. In addition, miR-1296 inversely regulated SRPK1 abundance by directly binding to its 3′-UTR, which subsequently resulted in suppression of p-AKT. Either SRPK1 re-expression or PI3K/AKT pathway activation, at least partially, abolished the effects of miR-1296 on migration, invasion and EMT progress of HCC cells. Furthermore, miR-1296 and SRPK1 expression were markedly correlated with adverse clinical features and poor prognosis of HCC patients. We showed that hypoxia was responsible for the underexpression of miR-1296 in HCC. And the promoting effects of hypoxia on metastasis and EMT of HCC cells were reversed by miR-1296.

**Conclusions:**

Underexpression of miR-1296 potentially serves as a prognostic biomarker in HCC. Hypoxia-induced miR-1296 loss promotes metastasis and EMT of HCC cells probably by targeting SRPK1/AKT pathway.

**Electronic supplementary material:**

The online version of this article (doi:10.1186/s12943-017-0675-y) contains supplementary material, which is available to authorized users.

## Background

Hepatocellular carcinoma (HCC) is the fifth most common malignancies worldwide and the second frequent cause of cancer-related death according to world health organization (WHO) data [1, 2]. Despite remarkable advances in diagnosis and improvement in therapeutic modalities, including novel chemotherapeutic interventions and target therapy, the long-term survival of HCC patients remains unsatisfactory due to the high rates of intrahepatic and distal metastasis [3, 4]. Therefore, it is critical to identify the potential molecular mechanisms underlying the progression and metastasis in HCC and thus provide novel therapeutic targets for cancer treatment.

MicroRNAs (miRNAs), a group of endogenous evolutionarily conserved non-coding small RNAs, act as post-transcriptional regulator of gene expression in cancer initiation, development and progression by binding to complementary sequences within the 3′-untranslated region (UTR) of target mRNA and subsequently inducing mRNA degradation or translational repression [5, 6]. Numerous evidences confirm that aberrantly expressed miRNAs play critical roles in multiple biological progresses in HCC [7–10], including cell proliferation, apoptosis, drug-resistance, metastasis and stem cell renewal, and have been identified as promising therapeutic and prognostic biomarkers in HCC diagnosis and treatment.

MiR-1296, a novel cancer-related miRNA, has been found to be dysregulated in cancers [11–13]. Zhu et al. demonstrated that miR-1296 was involved in the regulation of cell migration and invasion in human gastric cancer via targeting ERBB2/Rac1 signaling pathway [14]. MiR-1296 was significantly decreased in triple-negative breast cancer, and promoted cell cycle arrest and cisplatin sensitiveness of breast cancer cells [15]. Moreover, miR-1296 increased resistance to chemotherapeutic treatment and could be used as a new potential biomarker for breast cancer stem cell diagnosis [16]. These data suggest that miR-1296 plays a tumor suppressive role in malignancies. In addition, miR-1296 upregulation could serve as a predictive marker for the colon cancer cases with subsequent relapse [17], which indicates that miR-1296 serves as an oncogene in colon cancer. Taken together, the expression level and biological function of miR-1296 is cancer-specific. Nevertheless, the function of miR-1296 and its underlying molecular mechanisms in HCC remain unknown.

Epithelial-to-mesenchymal transition (EMT), is implicated in the invasion and metastasis of various cancers through transformation of adherent and polarized epithelial cells into an invasive mesenchymal cell phenotype [18, 19]. Moreover, the typical EMT process usually is characterized by decrease of the cell adhesion molecule E-cadherin and increase of Vimentin and N-cadherin expression. E-cadherin is an important determinant of epithelial cell-cell adhesion, while Vimentin and N-cadheirn are the mesenchymal markers [20, 21]. Increasing studies reveal that EMT is a main cause for HCC invasion and metastasis [22, 23]. However, the association between miR-1296 and EMT in HCC is poorly investigated.

Our results showed that underexpression of miR-1296 was associated with poor prognostic features of HCC patients. MiR-1296 inhibited migration, invasion and EMT progression of HCC cells in vitro and in vivo. Notably, serine-arginine protein kinase 1 (SRPK1) was identified as a direct target of miR-1296 and mediated the function of miR-1296 in HCC cells. In addition, miR-1296, SRPK1 and their combination were valuable predictors for the prognosis of HCC patients.

## Methods

### Clinical tissues

One hundred and twenty-six HCC tissues and matched adjacent non-tumor tissues were collected from Department of Hepatobiliary Surgery, the First Affiliated Hospital of Xi’an Jiaotong University during January 2009 to December 2011. Another cohort of ninety-eight HCC specimens were obtained from Department of Hepatobiliary Surgery, Sun Yat-Sen Memorial Hospital. Pathological diagnosis was performed according to the WHO criteria. The tissues were stored at −80 °C or embedded in paraffin. None of the patients received chemotherapy or radiotherapy before surgery. Written informed consent were obtained from all patients.

The human HCC cell lines including MHCC-97 L, HCCLM3, MHCC-97H, Huh7, Hep3B and the normal human immortalized normal hepatic cell line LO2 were purchased from the Institute of Biochemistry and Cell Biology (Chinese Academy of Sciences, Shanghai, China) and were cultured in complete Dulbecco’s modified Eagle’s medium (DMEM) (Invitrogen, Carlsbad, USA) containing 10% FBS (Invitrogen, Carlsbad, CA), 1% penicillin-streptomycin (Sigma, St. Louis, MO, USA) in a humidified atmosphere at 37 °C with 5% CO2.

### Quantitative reverse transcriptase polymerase chain reaction (qRT-PCR)

Total RNA from HCC tissues and cells was isolated using TRIzol reagent (Invitrogen, Carlsbad, CA) according to the manufacturer’s protocol. cDNA was reverse-transcribed from 2 μg total RNA using a Reverse Transcription Kit (Takara, Biochemical, Tokyo, Japan). cDNA was then amplified with a SYBR® Premix Ex Taq™ II (Perfect Real-Time) kit (Takara). The gene expression levels were calculated using the delta-delta Ct method with U6 or GAPDH as an internal control. Hsa-miR-1296 primer (HmiRQP0143), snRNA U6 qPCR Primer (HmiRQP9001), SRPK1 (HQP017724) and GAPDH (HQP006940) were purchased from Genecopoeia (Guangzhou, China).

### Cell transfection

MiRNA vectors, including precursor miR-1296 clones (HmiR0471), precursor miR-1296 scrambled control clones (miR-control; CmiR0001), miR-1296 inhibitors (anti-miR-1296; HmiR-AN0143) and miR-1296 inhibitor control clones (anti-miR-NC; CmiR-AN0001) were obtained from Genecopoeia (Guangzhou, China). The SRPK1 overexpression plasmid and specific siRNA against SRPK1 and a scramble siRNA were synthesized by Sangon Biotech Co., Ltd. (Shanghai, China). Cells were transfected with above vectors using Lipofectamine 2000 Reagent (Invitrogen Life Technologies) in accordance with the manufacturer’s protocol.

### Western blot analysis

The whole proteins were lysed in RIPA buffer supplemented with protease and phosphatase inhibitors (Roche) and the concentrations were quantified with BCA Protein Assay Kit (Tiangen, Beijing, China), and an equal amount of 40 μg protein was separated by 10% SDS-PAGE gel and then transferred onto PVDF membranes (Millipore, Billerica, MA, USA). The membranes were blocked with 5% nonfat milk in TBST for 2 h at room temperature and incubated overnight with specific primary antibodies at 4 °C. Then the membranes were washed three times by TBST and incubated with HRP-conjugated secondary antibody for 2 h at room temperature (ZSGB-BIO, China). Detection was performed by enhanced chemiluminescence kit (Amersham, Little Chalfont, UK). GAPDH (G8140; US Biological, Swampscott, MA, USA) was used as protein loading control. The SRPK1 primary antibody was obtained from Abcam (Cambridge, MA, USA). The antibodies against E-cadherin, N-cadherin, Vimentin, AKT, p-AKT, ZO-1, ZEB1, Slug, Snail, Twist, ERBB2, CCND1 and MCM2 were purchased from Cell Signaling Technology (Beverly, MA, USA).

### Immunofluorescence (IF)

HCC cells that transfected with corresponding miRNA vectors were seeded on chamber slides and were fixed with 4% paraformaldehyde for 10 min at room temperature. Then, cells were incubated with antibodies against E-cadherin (Cell Signaling Technology) or Vimentin (Cell Signaling Technology) at 4 °C overnight. Then, the slides were incubated with matched secondary antibodies (Invitrogen) at room temperature for 1 h. The nuclear of EC cells were stained with DAPI (Sigma) at room temperature for 10 min. Fluorescence confocal images were captured using a LSM 5 Pascal Laser Scanning Microscope (Zeiss Germany, Oberkochen, Germany).

### Cell migration and invasion analyses

Matrigel-uncoated and -coated transwell inserts (8 μm pore size; Millipore) were used to evaluate cell migration and invasion. Briefly, 2 × 10^4^ transfected cells were suspended in 150 μL serum free DMEM medium into the upper chamber, and 700 μL DMEM medium containing 20% FBS was placed in the lower chamber. After 24 h incubation, cells were fixed in 4% paraformaldehyde for 20 min and stained with 0.1% crystal violet dye for 15 min. The cells on the inner layer were softly removed with a cotton swab and counted at five randomly selected views, and the average cell number per view was calculated.

### Immunohistochemistry (IHC) analysis

Briefly, 4 μm sections were deparaffinized in xylene, rehydrated through graded ethanols, followed by blocking of endogenous peroxidase activity in 3% hydrogen peroxide for 10 min at room temperature. The corresponding antibody (1:300, Cell Signaling Technology, Inc.) was applied as the primary antibody by a streptavidin peroxidase-conjugated (SP-IHC) method. The staining results were semi-quantitatively evaluated by the multiply of staining intensity and the percentage of positive staining cells. The percentage of positive cells was given into four grades: 0 for <5%; 1 for 6%–25%; 2 for 26%–50%; 3 for 51%–75% and 4 for >75%. Staining intensity was assessed by four degrees: 0, negative; 1, weak; 2, moderate; and 3, strong. Each section was assayed for ten independent high magnifications (×400) fields to get the average scores.

### Luciferase reporter assay

The 3′-UTR sequence of SRPK1 predicted to interact with miR-1296, together with a corresponding mutated sequence within the predicted target sites, were synthesized and inserted into the pmiR-GLO dual-luciferase miRNA target expression vector (Promega, Madison, WI, USA) called wt-SRPK1 3′-UTR and mt-SRPK1 3′-UTR. Subsequently, HCCLM3 or Hep3B cells that were plated into 24-well plate and were transfected with corresponding vectors. Cells were co-transfected with the wild-type or mutant 3′-UTR of SRPK1 vector using the Lipofectamine 2000 reagent (Invitrogen, USA). After 48 h, cells were harvested and measured according to the manufacturer’s instructions (Dual-Luciferase Assay System; Promega). pRL-TK expressing Renilla luciferase was cotransfected as an internal control to correct the differences in both transfection and harvest efficiencies.

### In vivo experiments

4–6 week-old female BALB/c nude mice (Centre of Laboratory Animals, The Medical College of Xi’an Jiaotong University, Xi’an, China) were randomized into two groups (*n* = 5), and either HCCLM3-miR-1296 or HCCLM3-miR-control cells (1 × 10^6^); Hep3B-anti-miR-1296 or Hep3B-anti-miR-NC were injected into the tail veins for the establishments of pulmonary metastatic model. Mice were sacrificed 10 weeks’ post injection and examined microscopically by hematoxylin and eosin (H&E) staining for the development of lung metastatic foci. Animals were housed in cages under standard conditions. The protocols for these animal experiments were approved by the Ethics Review Committee of Xi’an Jiaotong University.

### Statistical analysis

Data are presented as the mean ± SD and performed at least three independent replicates. SPSS software, 16.0 (SPSS, Inc., Chicago, IL, USA) and Graphpad Prism 6.0 (CA, USA) were used for a two-tailed Student t-test, Pearson’s correlation analysis, Kaplan-Meier method and the log-rank test to evaluate the statistical significance. Differences were defined as *P* < 0.05.

## Results

### Reduced miR-1296 expression confers metastasis and recurrence of HCC

**Fig. 1:**
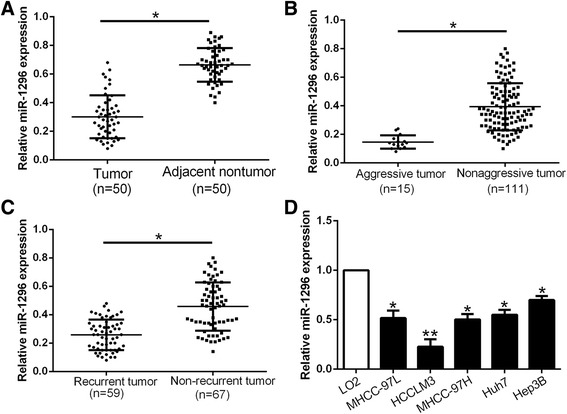
miR-1296 is underexpressed in HCC. Comparing differences in the expressions of miR-1296 between (**a**) HCC and matched tumor-adjacent tissues; (**b**) aggressive and nonaggressive tumor tissues; (**c**) HCC tissues arising from recurrent and non-recurrent groups; and (**d**) HCC cell lines and the immortalized hepatic cell line LO2. *P < 0.05, **P < 0.01

We determine the expression level of miR-1296 in 50 pairs of randomly selected tumor tissues and matched adjacent non-tumor tissues. The expression of miR-1296 in HCC tissues was significantly lower than that in matched adjacent non-tumor tissues (*P* < 0.05, Fig. 1a). We defined the HCC tissues with intrahepatic metastasis, tumor invasion into bile duct and venous infiltration as aggressive HCC tissues. We found that miR-1296 was obviously decreased in aggressive HCC tissues compare to non-aggressive tissues (*P* < 0.05, Fig. 1b). Moreover, miR-1296 was down-regulated in tumor tissues from patients with recurrence compared to patients without recurrence (*P* < 0.05, Fig. 1c). In addition, the miR-1296 expression was significantly down-regulated in all HCC cell lines as compared with normal hepatic cell line LO2 (*P* < 0.05, respectively, Fig. 1d). Therefore, reduced expression of miR-1296 is probably correlated with metastasis and recurrence of HCC.

### miR-1296 inhibits HCC cell migration and invasion in vitro

**Fig. 2:**
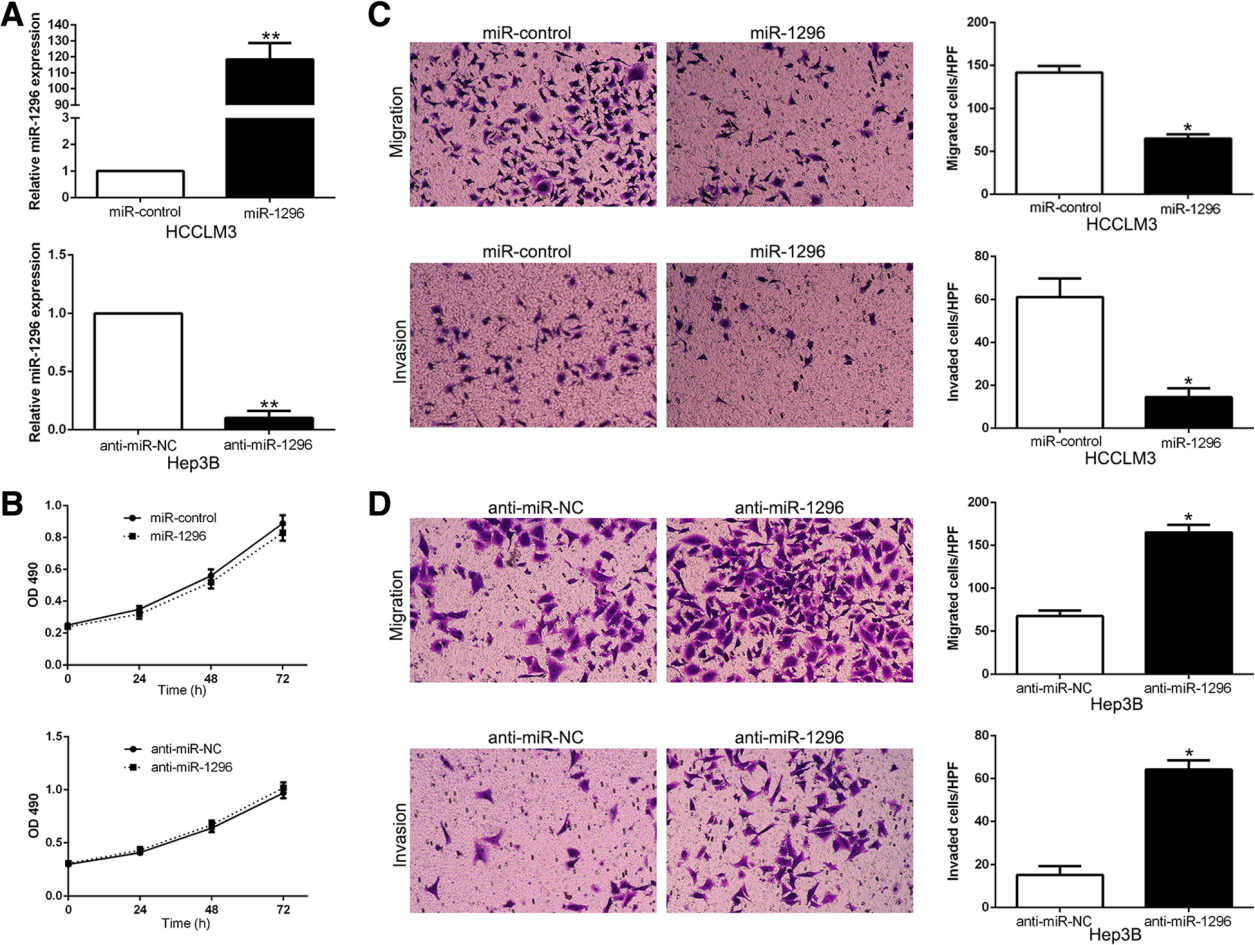
miR-1296 inhibits the migration and invasion of HCC cells in vitro. **a** HCCLM3 and Hep3B cells that were transfected with corresponding miRNA vectors were subjected to qRT-PCR for miR-1296 expression. **b** Modulating miR-1296 expression showed no obvious effect on proliferation of HCC cells, as determined by MTT assays. **c** Cell migration and invasion as measured by Transwell assays were inhibited by miR-1296 overexpression in HCCLM3 cells. **d** miR-1296 knockdown promoted migration and invasion of Hep3B cells. *P < 0.05, **P < 0.01

To investigate the biological function of miR-1296 in HCC, gain- and loss-of-function experiments were performed in HCCLM3 and Hep3B cells, respectively, which showed different levels of endogenous miR-1296. As measured by qRT-PCR, we confirmed that miR-1296 was effectively overexpressed in HCCLM3 cells while knocked down in Hep3B cells (*P* < 0.05, Fig. 2a). Firstly, we assessed cell growth by MTT assays and no significant difference was observed after modulating miR-1296 expression in HCC cells (Fig. 2b). Next, transwell assays revealed that miR-1296 overexpression significantly inhibited the migration and invasion of HCCLM3 cells (*P* < 0.05, respectively, Fig. 2c), whereas miR-1296 knockdown obviously increased the number of migrated and invaded Hep3B cells (*P* < 0.05, respectively, Fig. 2d). These results suggest that miR-1296 regulates HCC cell migration and invasion.

### miR-1296 inhibits EMT process of HCC cells

**Fig. 3:**
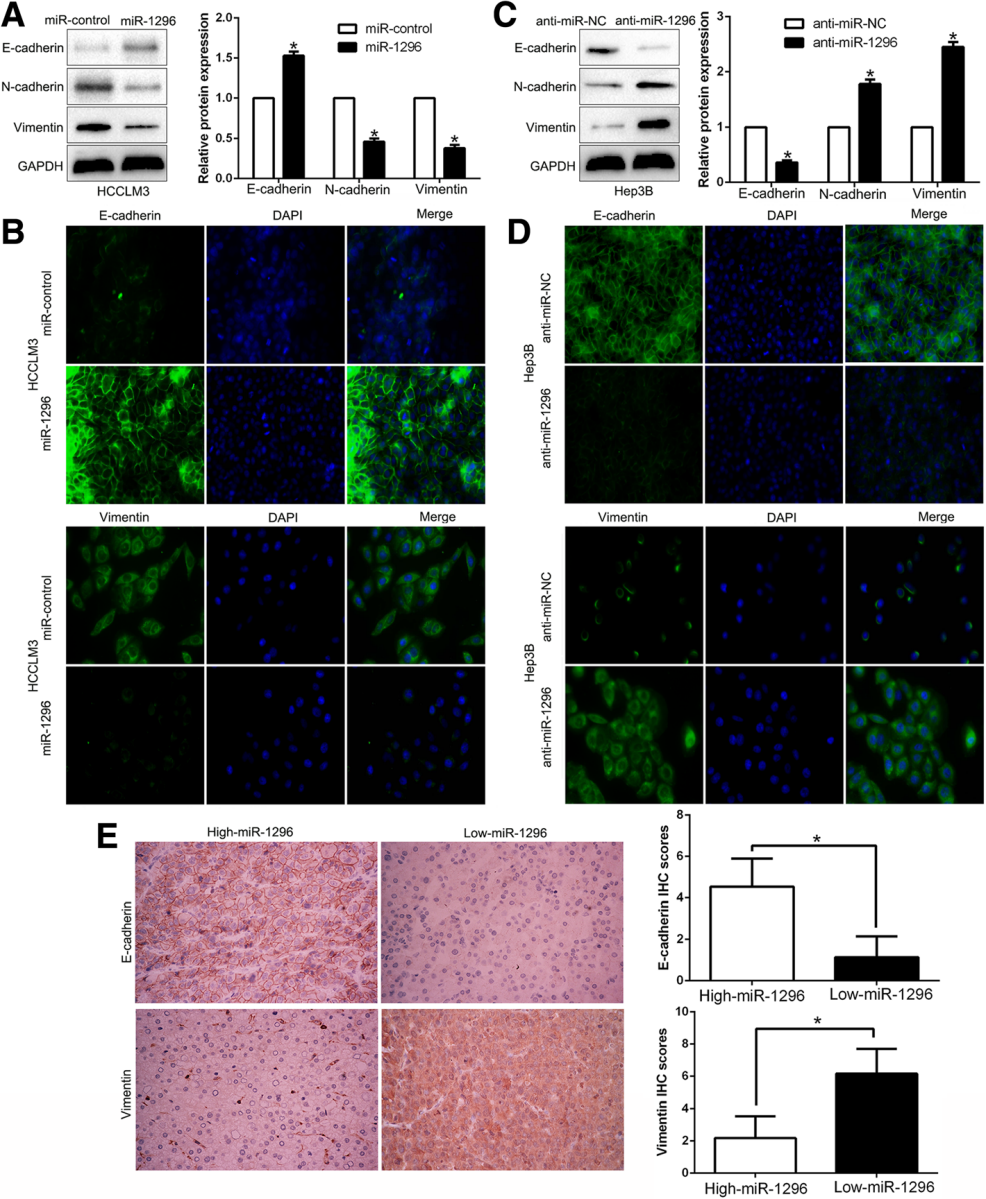
miR-1296 inhibits the EMT process of HCC cells. **a** Western blot analysis of epithelial marker E-cadherin, and mesenchymal markers N-cadherin and Vimentin after miR-1296 overexpression in HCCLM3 cells. **b** IF staining of E-cadherin and Vimentin after miR-1296 overexpression in HCCLM3 cells. **c** miR-1296 knockdown decreased E-cadherin expression, and increased the levels of N-cadherin and Vimentin in Hep3B cells. **d** IF staining of E-cadherin and Vimentin after miR-1296 knockdown in Hep3B cells. **e** Immunohistochemistry of E-cadherin and Vimentin were showed and compared between miR-1296 high expressing HCC tissues and miR-1296 low expressing cases. *P < 0.05

EMT has been recognized as a critical regulator in the initiation of HCC metastasis [24]. Further experiments were performed to disclose whether miR-1296 inhibited HCC metastasis via regulating EMT. Western blot and IF results showed that miR-1296 overexpression increased the expression epithelial marker E-cadherin and inhibited the levels of mesenchymal marker N-cadherin and Vimentin in HCCLM3 cells (*P* < 0.05, respectively, Fig. 3a and b). In contrast, miR-1296 knockdown decreased E-cadherin expression and increased N-cadherin and Vimentin expression in Hep3B cells (*P* < 0.05, respectively, Fig. 3c and d). Moreover, we also determined other EMT markers and observed the similar results (*P* < 0.05, Additional file 1: Figure S1). In addition, we further explored the correlation between the expression of miR-1296 and EMT markers in HCC tissues. We found that E-cadherin expression in miR-1296 high expressing HCC tissues was notably higher than that in low expressing cases (*P* < 0.05, Fig. 3e and Additional file 2: Figure S2). Conversely, the expression of Vimentin in the miR-1296 high expression group was markedly lower than that in low expression group (*P* < 0.05, Fig. 3e and Additional file 2: Figure S2). Taken together, these results suggest that miR-1296 suppresses EMT process of HCC cells.

### SRPK1 is a direct target of miR-1296 in HCC

**Fig. 4:**
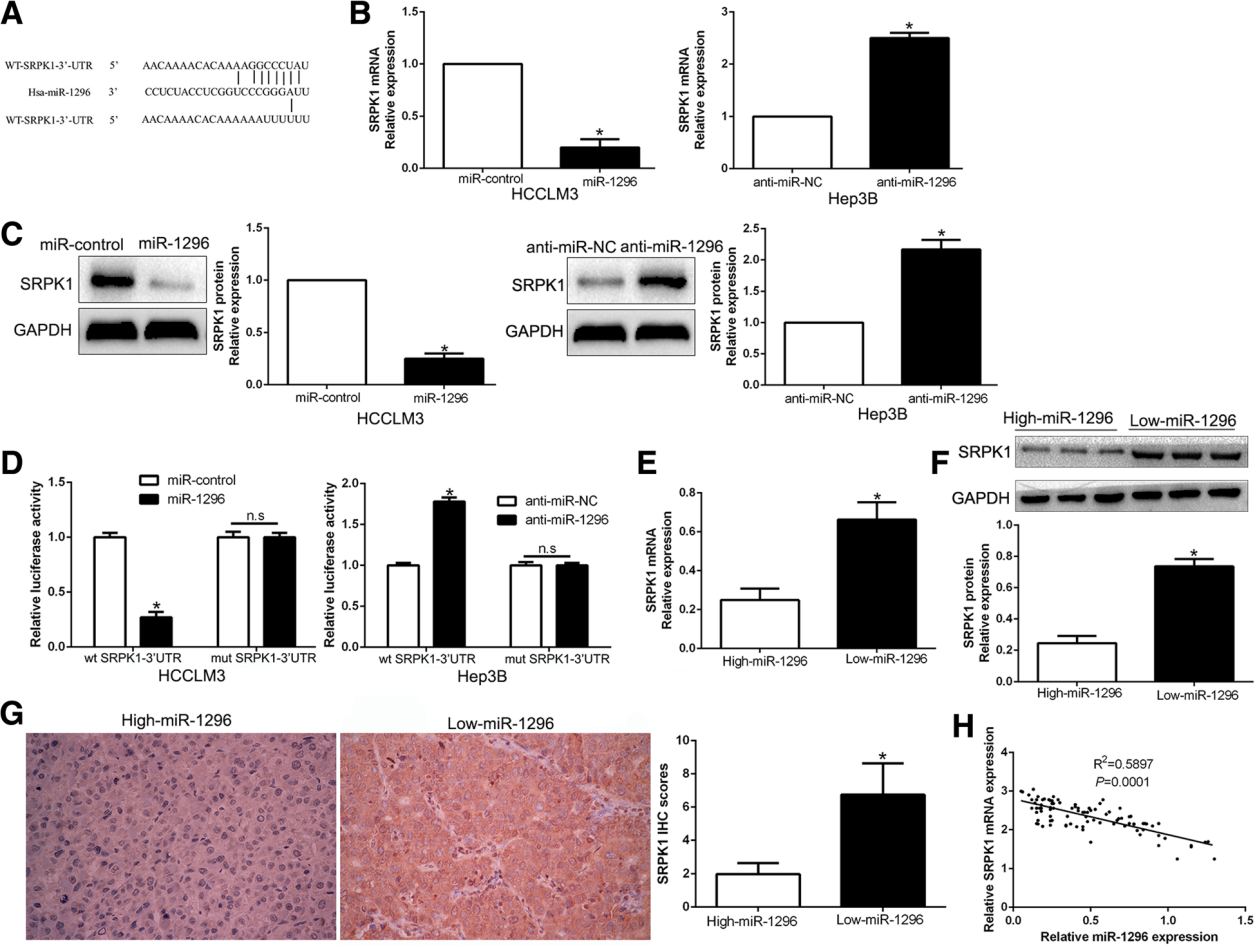
SRPK1 is a direct target of miR-1296 in HCC cells. **a** miR-1296 and its putative binding sequences in the 3′-UTR of SRPK1. The mutant binding site was generated in the complementary site for the seed region of miR-1296. **b** HCCLM3 and Hep3B cells that were transfected with precursor miR-1296 and miR-1296 inhibitors (anti-miR-1296), respectively, were subjected to qRT-PCR for SRPK1 mRNA expression. **c** miR-1296 overexpression reduced the expression of SRPK1 protein in HCCLM3 cells and miR-1296 knockdown increased the level of SRPK1 protein in Hep3B cells. **d** miR-1296 overexpression significantly suppressed, while miR-1296 loss increased the luciferase activity that carried wild-type (wt) but not mutant (mt) 3′-UTR of SRPK1. **e** and **f** The expression of SRPK1 in miR-1296 high-expressing tumors was significantly lower than that in miR-1296 low-expressing tumors, as determined by qRT-PCR and immunoblotting. **g** Representative immunohistochemical staining showed a weak staining of SRPK1 in miR-1296 high-expressing HCC tissue and strong staining of SRPK1 in the miR-1296 low-expressing tumor. **h** An inverse correlation between the levels of miR-1296 and SRPK1 mRNA was observed in HCC tissues. *P < 0.05

To elucidate the molecular mechanisms by which miR-1296 exerts its functional effects on HCC cells, we predicted potential targets by different miRNA target algorithms (TargetScan, miRanda and miRbase) and found conserved putative miR-1296 binding sites at the 3′-UTR of SRPK1 (Fig. 4a). Previous studies have demonstrated that SRPK1 is highly expressed in HCC cells and tissues, and associates with poor prognosis and aggressive phenotype of HCC [25, 26]. To confirm this hypothesis, we performed qRT-PCR and Western blot analysis and found that ectopic expression of miR-1296 dramatically decreased, whereas miR-1296 knockdown increased the expressions of SRPK1 mRNA and protein in HCC cells (*P* < 0.05, respectively, Fig. 4b and c). However, modulating miR-1296 expression in HCC cells didn’t affect the levels of ERBB2, CCND1 and MCM2 protein, which were confirmed as direct targets of miR-1296 in other tumors (Additional file 3: Figure S3). Next, luciferase reporter assay confirmed that miR-1296 overexpression significantly decreased the luciferase activity of wild-type (wt) SRPK1 3′-UTR (*P* < 0.05, Fig. 4d). In contrast, miR-1296 knockdown increased the luciferase activity of wt SRPK1 3′-UTR (*P* < 0.05, Fig. 4d). But modulating miR-1296 expression showed no significant effect on the luciferase activity of mutant (mt) SRPK1 3′-UTR (Fig. 4d). Moreover, we found the expressions of SRPK1 in the miR-1296 high-expressing tumors were significantly lower than those in the miR-1296 low-expressing tumors (*P* < 0.05, respectively, Fig. 4e-g). Notably, an obvious inverse correlation between the levels of miR-1296 and SRPK1 mRNA was revealed by Spearman’s correlation analysis in HCC tissues (*P* < 0.05, Fig. 4h). Taken together, these data indicate that SRPK1 is a downstream of miR-1296 in HCC.

### SRPK1 mediates the effects of miR-1296 on HCC cells

Previous studies confirmed that SRPK1 predicts poor survival and promotes cell proliferation of HCC through PI3K/AKT signaling [25, 26]. Subsequently, to confirm the biological role of SRPK1 in metastasis and EMT progression, SRPK1 was knocked down by a specific siRNA in HCCLM3 cells (*P* < 0.05. Additional file 4: Figure S4A) and SRPK1 silencing led to a significant reduction of cell migration and invasion (*P* < 0.05. Additional file 4: Figure S4B). Moreover, Hep3B cells were transfected with empty vector (EV) or SRPK1 plasmid (*P* < 0.05. Additional file 4: Figure S4C). We demonstrated that SRPK1 overexpression prominently promoted migration and invasion of Hep3B cells (*P* < 0.05. Additional file 5: Figure S4D). In accordance, SRPK1 positively regulated EMT process of HCC cells (*P* < 0.05. Additional file 4: Figure S4E and 4F). These data suggest that SRPK1 alteration could mimic miR-1296-induced metastasis and EMT process in HCC cells.

**Fig. 5:**
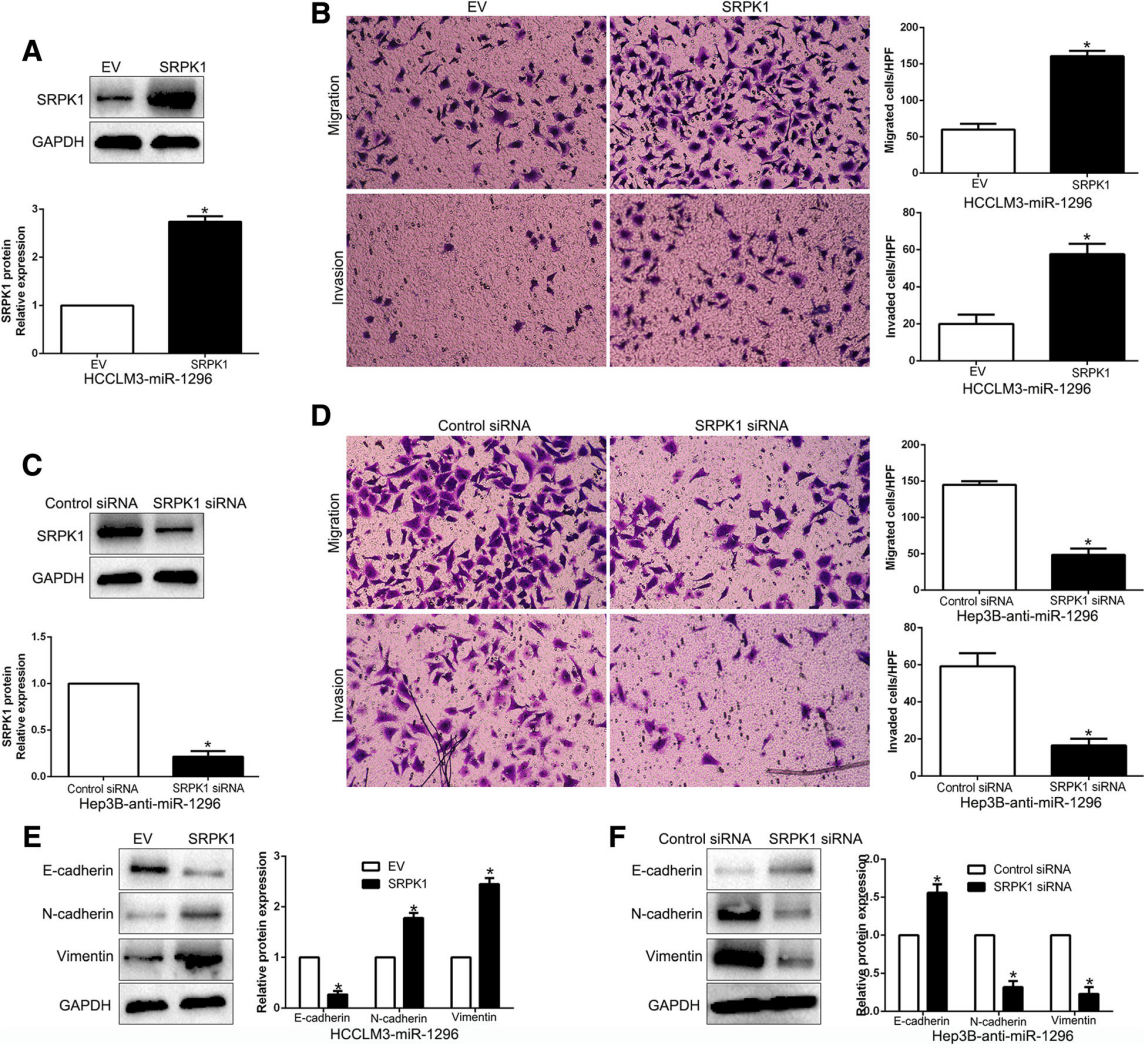
Modulation of SRPK1 partially abolishes miR-1296-mediated cellular processes in HCC. **a** miR-1296-overexpressing HCCLM3 cells that were transfected with empty vector (EV) or SRPK1 overexpression plasmid were subjected to western blot for SRPK1. **b** SRPK1 restoration promoted migration and invasion of miR-1296-overexpressing HCCLM3 cells. **c** miR-1296-suppressive Hep3B cells that were transfected with scrambled siRNA or SRPK1 siRNA were subjected to western blot for SRPK1. **d** SRPK1 knockdown abrogated the effects of miR-1296 loss on migration and invasion of Hep3B cells. **e** SRPK1 restoration decreased the expression of E-cadherin and increased the levels of N-cadherin and Vimentin in miR-1296 overexpressing HCCLM3 cells. **f** SRPK1 knockdown abolished the effects of miR-1296 loss on EMT process of Hep3B cells. *P < 0.05

To confirm that SRPK1 is a functional mediator of miR-1296, SRPK1 was restored by overexpression plasmids in miR-1296-overexpressing HCCLM3 cells (*P* < 0.05, Fig. 5a). SRPK1 restoration abrogated the inhibitory effects of miR-1296 on migration and invasion of HCCLM3 cells (*P* < 0.05, respectively, Fig. 5b). Similarly, SRPK1 knockdown by a specific siRNA in miR-1296-suppressive Hep3B cells significantly reversed the promoting function induced by miR-1296 loss on Hep3B cell migration and invasion (*P* < 0.05, respectively, Fig. 5c and d). Furthermore, modulating SPRK1 expression mediated the effects of miR-1296 on EMT events in HCC cells (*P* < 0.05, respectively, Fig. 5e and f). These data demonstrated that SRPK1 is not only a downstream target, but also a functional mediator of miR-1296 in HCC.

### miR-1296 ameliorates the metastatic potential of HCC cells in mice

**Fig. 6:**
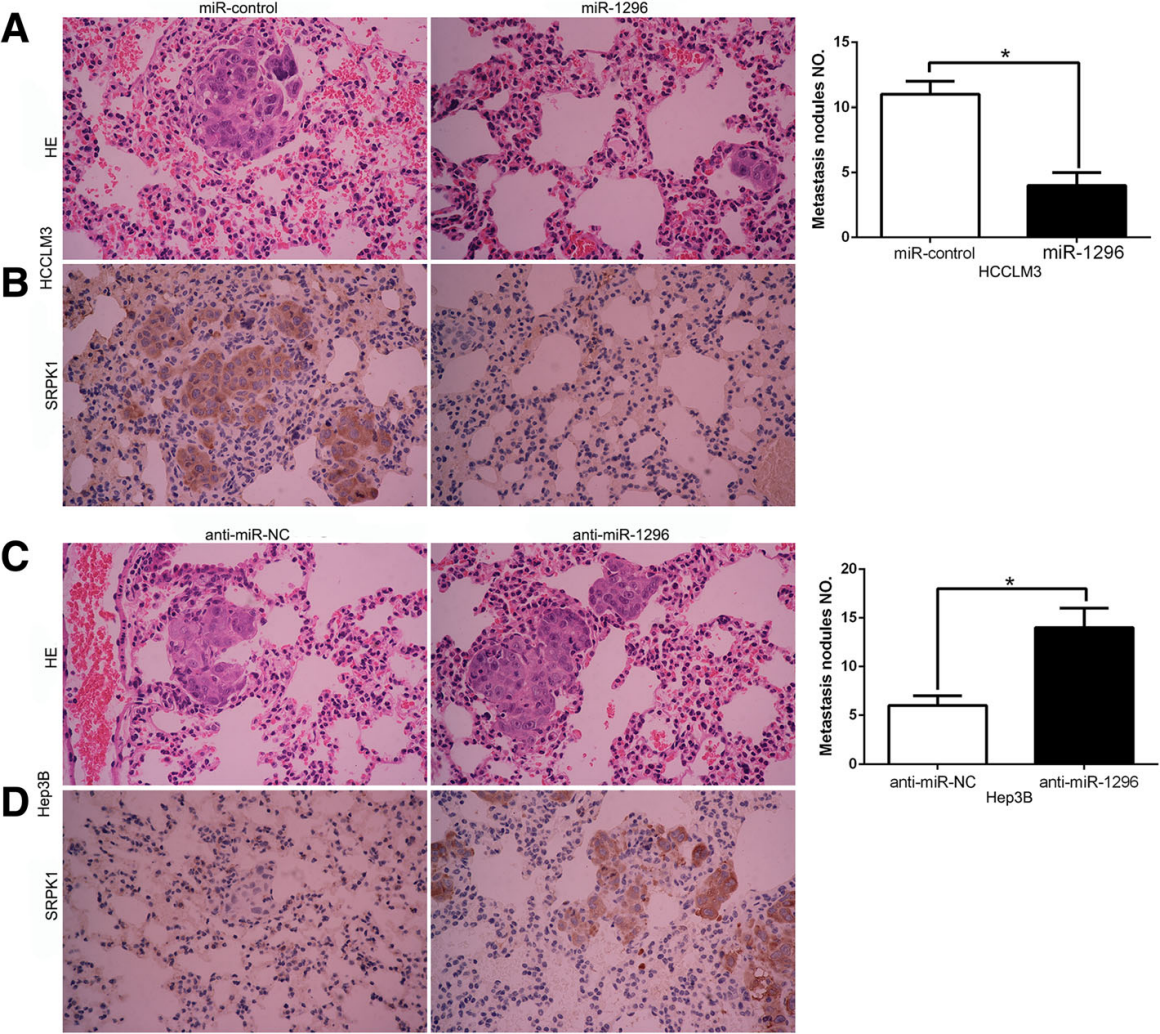
miR-1296 inhibits the lung metastasis of HCC cells in nude mice. **a** Representative HE staining of lung metastases between HCCLM3-miR-1296 cells and control cells. **b** Immunohistochemistry suggested that SRPK1 expression showed a weaker staining in HCCLM3-miR-1296 cells than control cells. **c** Representative HE staining of lung metastases between Hep3B-anti-miR-1296 cells and control cells. **d** Immunohistochemistry revealed that SRPK1 expression in shows a stronger staining in Hep3B-anti-miR-1296 cells than control cells. *P < 0.05

To further confirm biological function of miR-1296 in vivo, miR-1296 overexpressing HCCLM3 cells (HCCLM3-miR-1296) and control cells (HCCLM3-miR-control) or miR-1296 silencing Hep3B cells (Hep3B-anti-miR-1296) and control cells (Hep3B-anti-miR-NC) were injected into the lateral veins of nude mice. The results showed that miR-1296 overexpression group showed fewer and smaller foci in the lungs of nude mice via microscopic evaluation (*P* < 0.05, Fig. 6a). Moreover, we also demonstrated that lung sections of miR-1296 overexpression group in fact showed decreased expression of SRPK1 (*P* < 0.05, Fig. 6b). In contrary, miR-1296 knockdown in Hep3B cells led to a significant increased lung metastasis nodules and SRPK1 expression (*P* < 0.05, Fig. 6c and d). Taken together, these data suggest that miR-1296 inhibits metastatic behaviors of HCC and regulates SRPK1 expression in vivo.

### Clinical significance of miR-1297 and SRPK1 expression for HCC patients

**Fig. 7:**
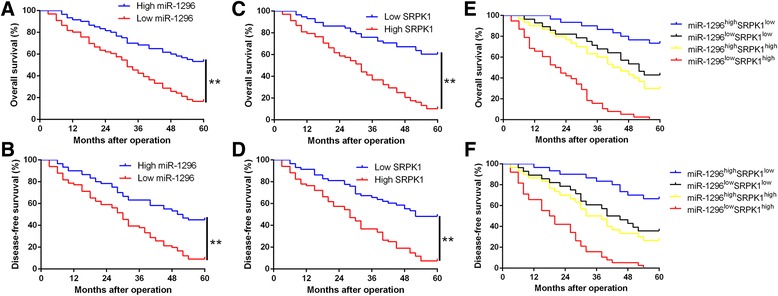
The prognostic value of miR-1296 and SRPK1 for HCC patients. **a** and **b** Overall survival (OS) and disease-free survival (DFS) were compared between miR-1296 high expressing HCC patients and low expressing cases. **c** and **d** OS and DFS were compared between SRPK1 high expressing HCC patients and low expressing cases. **e** and **f** OS and DFS were compared between four subgroups of HCC patients (subgroup I: high miR-1296/low SRPK1; subgroup II: low miR-1296 /low SRPK1; subgroup III: high miR-1296/high SRPK1; subgroup IV: low miR-1296/high SRPK1). For each cohort, subgroups were divided according to the cutoff values, which were determined as the median level of miR-1296 and SRPK1 in HCC tissues. **P < 0.01

Above results confirmed the biological role of miR-1296 and SRPK1 in HCC. We next explored the clinical significance of miR-1296 and SRPK1 in HCC patients. For each cohort, subgroups were divided according to the cutoff values, which were determined as the median level of miR-1296 and SRPK1 in HCC tissues. As shown in Table 1, we found that underexpression of miR-1296 was significantly associated with tumor-node-metastasis (TNM) stage (III + IV, *P* = 0.011), venous invasion (*P* = 0.024) and multiple tumor nodes (*P* = 0.012), while SRPK1 overexpression was correlated with venous invasion (*P* = 0.006) and advanced TNM stage (*P* = 0.001). These data indicate that aberrant expressions of miR-1296 and SRPK1 is correlated with poor prognostic features of HCC patients. Moreover, Kaplan-Meier survival curves suggested that miR-1296 low expressing HCC patients showed a notably reduced overall survival (OS) and disease-free survival (DFS), while SRPK1 high expressing patients conferred an obviously poorer OS and DFS (*P* < 0.05, respectively, Fig. 7a-d). With combination analysis, the data showed that patients with low miR-1296 and high SRPK1 expression had the worst OS and DFS (*P* < 0.05, respectively, Fig. 7e and f). Moreover, we confirmed the similar results in another cohort of HCC patients (*P* < 0.05, Additional file 5: Figure S5). These data suggest that combination of miR-1296 and SRPK1 is a potential biomarker for the clinical outcome of HCC patients.

**Table 1.** Correlation between the clinicopathologic characteristics and miR-1296 and SRPK1 expression in HCC (*n* = 126)

Clinical parameters	Cases	Expression level		*P* value	Expression level		*P* value
		MiR-1296^high^ (*n*=60)	MiR-1296^low^ (*n*=66)		SRPK1^high^ (*n*=68)	SRPK1^low^ (*n*=58)	
Age(years)							
<65	67	31	36	0.746	37	30	0.763
≥65	59	29	30		31	28	
Gender							
Male	100	49	51	0.543	55	45	0.649
Female	26	11	15		13	13	
Tumor size (cm)							
<5	95	46	49	0.752	50	45	0.598
≥5	31	14	17		18	13	
Tumor number							
Solitary	107	56	51	0.012*	56	51	0.383
Multiple	19	4	15		12	7	
Edmondson							
I+II	89	43	46	0.808	47	42	0.686
III+IV	37	17	20		21	16	
TNM stage							
I+II	99	53	46	0.011*	46	53	0.001*
III+IV	27	7	20		22	5	
Capsular							
Present	85	41	44	0.842	43	42	0.273
Absent	41	19	22		25	16	
Venous invasion							
Present	12	2	10	0.024*	11	1	0.006*
Absent	114	58	56		57	57	
AFP (ng/mL)							
<400	39	17	22	0.544	23	16	0.450
≥400	87	43	44		45	42	
HBsAg							
Positive	116	56	60	0.615	61	55	0.466
Negative	10	4	6		7	3	

HCC, hepatocellular carcinoma; AFP, alpha-fetoprotein; TNM, tumor-node-metastasis. *Statistically significant

### PI3K/AKT signaling is essential for the biological function of miR-1296 in HCC

**Fig. 8:**
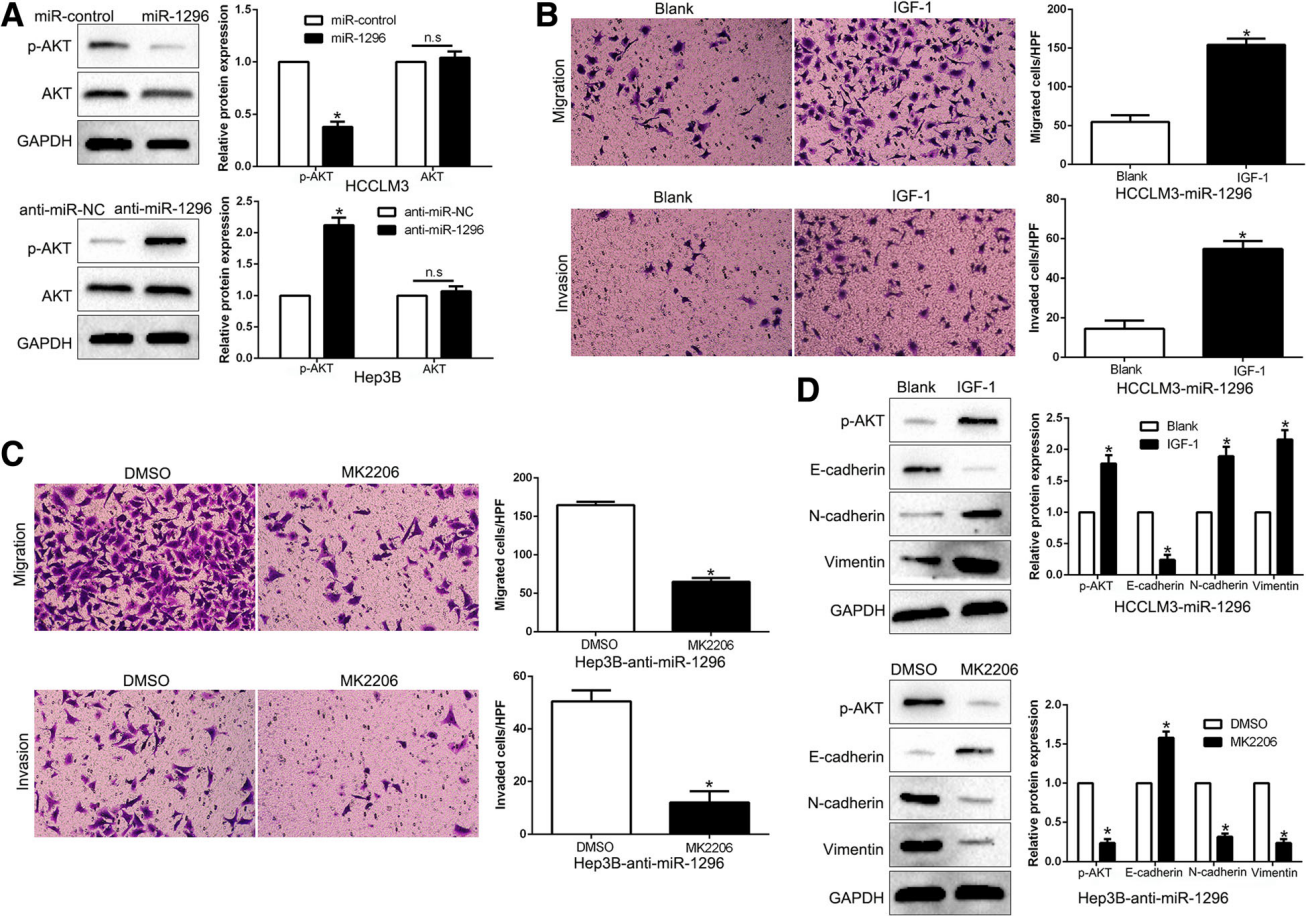
PI3K/AKT signaling is essential for the biological function of miR-1296 in HCC. **a** HCCLM3 and Hep3B cells that were transfected with corresponding miRNA vectors were subjected to immunoblotting for phosphorylated AKT and AKT. **b** IGF-1 treatment promoted the migration and invasion of miR-1296 overexpressing HCCLM3 cells. **c** AKT inhibitor MK2206 treatment abrogated the effect of miR-1296 loss on mobility of Hep3B cells. **d** Western blot analysis indicated that modulating AKT phosphorylation reversed the effects of miR-1296 alteration on EMT process of HCC cells. *P < 0.05

Previous study finds that SRPK1 promotes the activation of PI3K/AKT signaling and plays a key role in the invasion and metastasis of HCC [26]. As shown in Fig. 8a, miR-1296 overexpression significantly decreased, while miR-1296 knockdown increased the level of phosphorylated AKT in HCC cells (*P* < 0.05, respectively). These data indicate that miR-1296 suppresses the activation of PI3K/AKT pathway in HCC cells. Next, we treated miR-1296-overexpressing HCCLM3 cells with insulin-like growth factor 1 (IGF-1), which was an activator of PI3K/AKT pathway. We found that IGF-1 treatment at least partially rescued the miR-1296-induced inhibition of cell migration and invasion (*P* < 0.05, respectively, Fig. 8b). Conversely, the restraint of the PI3K/AKT pathway by MK2206 abrogated the effects of miR-1296 knockdown on Hep3B cell migration and invasion (*P* < 0.05, respectively, Fig. 8c). Moreover, modulating activation of PI3K/AKT pathway reversed the regulatory effects of miR-1296 on EMT events of HCC cells (*P* < 0.05, respectively, Fig. 8d). Thus, our results demonstrate that PI3K/AKT signaling functions in miR-1296-regulated HCC cell mobility and EMT.

### miR-1296 is down-regulated in hypoxia condition and mediates the effects of hypoxia on HCC metastasis

**Fig. 9:**
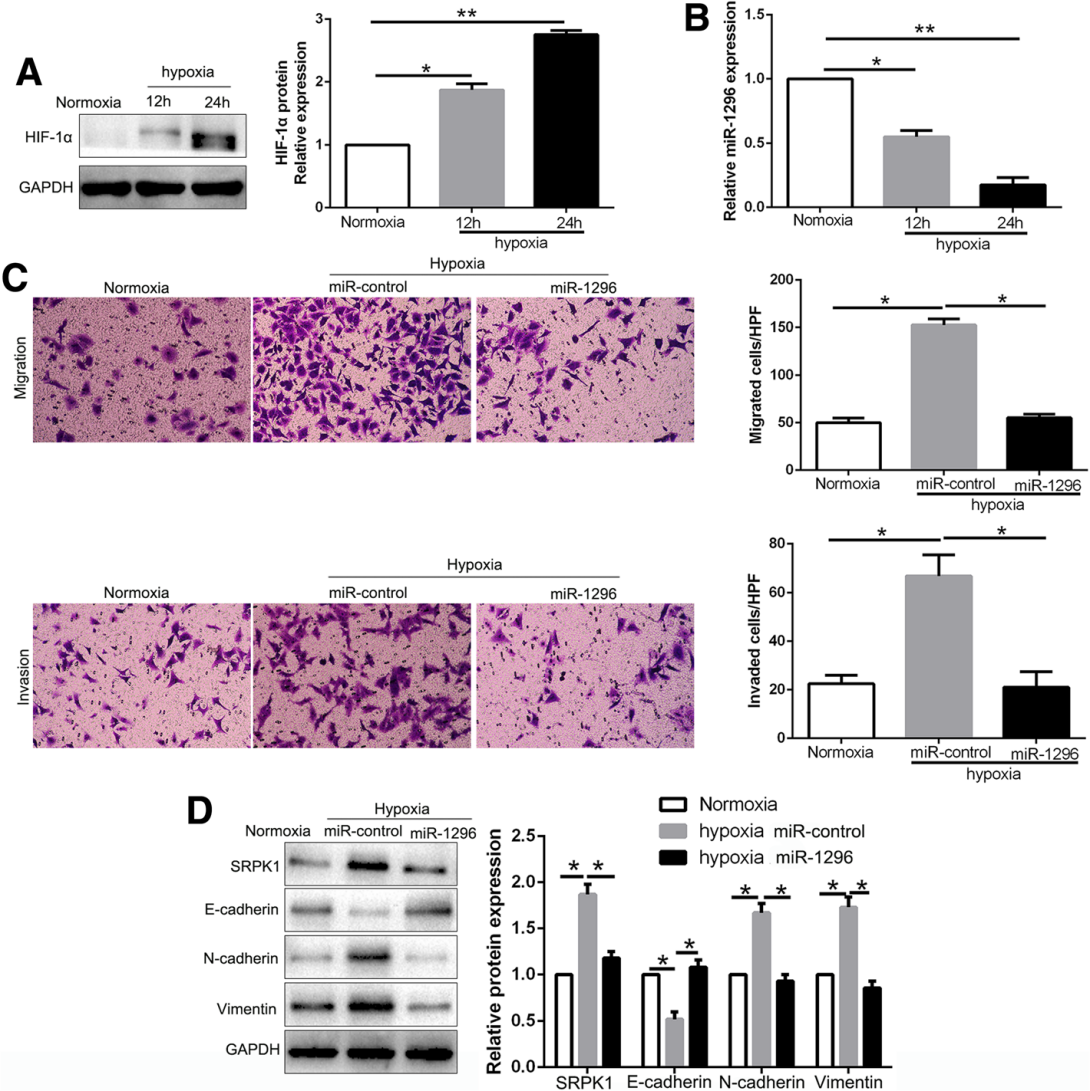
miR-1296 mediates the promoting effects of hypoxia on metastasis and EMT of HCC cells. **a** The expressions of HIF-1α in different time points in normoxia and hypoxia condition. **b** The levels of miR-1296 in Hep3B cells cultured in normoxia and hypoxia. **c** Transwell assays revealed that hypoxia promoted migration and invasion of Hep3B cells, while miR-1296 overexpression abolished the effects of hypoxia. **d** Hypoxia facilitated the EMT process of Hep3B cells and miR-1296 restoration showed a opposite effect. *P < 0.05, **P < 0.01

We further explored the reason that caused the decrease of miR-1296 in HCC. Previous studies confirm that hypoxia is a prevalent tumor microenvironment as a result of an imbalance between oxygen supply and consumption in rapidly growing tumor, and plays a critical role in cancer metastasis [27]. Notably, SRPK1, a direct downstream target of miR-1296 in this study, is elevated at mRNA and protein level in hypoxia condition [28]. Moreover, the effects of SRPK1 knockdown on cell growth, migration and invasion in glioma could be reversed in hypoxia [29]. Thus, we hypothesized that the miR-1296-SRPK1 axis could be regulated by hypoxia. Hypoxia condition significantly increased HIF-1α expression in Hep3B cells (*P* < 0.05, Fig. 9a) and led to a decrease of miR-1296 expression (*P* < 0.05, Fig. 9b). Interestingly, miR-1296 overexpression abolished the promoting effects of hypoxia on migration and invasion of Hep3B cells (*P* < 0.05, Fig. 9c). Similarly, the positive effects of hypoxia on EMT process were reversed by miR-1296 restoration in Hep3B cells (*P* < 0.05, Fig. 9d). Furthermore, to confirm that the role of SRPK1/AKT pathway in hypoxia condition, we determined the expression of SRPK1 and AKT expression in hypoxia condition. We found that hypoxia condition significantly increased SRPK1 and p-AKT expression in Hep3B cells (*P* < 0.05, Additional file 6: Figure S6A). Moreover, SRPK1 knockdown by a specific siRNA or p-AKT inhibitor MK2206 abolished the promoting effects of hypoxia on migration and invasion of Hep3B cells (*P* < 0.05, Additional file 6: Figure S6B and C). Similarly, the positive effects of hypoxia on EMT process were reversed by SRPK1 knockdown or p-AKT inhibition in Hep3B cells (*P* < 0.05, Additional file 6: Figure S6D). In conclusion, these results indicated that miR-1296 loss functions in hypoxia-induced migration, invasion and EMT events of HCC cells.

## Discussion

Numerous studies demonstrated that aberrantly expressed miRNAs were involved in the cancer initiation, development and progression, including HCC [30]. MiRNAs have been identified as novel prognostic biomarkers and effective therapeutic targets of HCC [31]. In this research, we found that miR-1296 was significantly down-regulated in HCC tissues and cell lines for the first time. Moreover, the aggressive and recurrent phenotype of HCC showed a lower expression of miR-1296. These data indicate that miR-1296 plays a tumor suppressive role in HCC.

Local and systemic metastasis is a major cause leading to a dismal prognosis of HCC. Increasing evidences confirm that miRNAs are recognized as key regulators in metastasis of cancers, including HCC [32, 33]. In this study, gain- and loss-of-function experiment confirmed that miR-1296 overexpression inhibited the migration and invasion of HCC cells while miR-1296 knockdown increased these metastatic behaviors in vitro and in vivo. EMT is a critical process in the invasion and metastasis of HCC [34]. In present study, we demonstrated that miR-1296 suppressed EMT events of HCC cells. Moreover, we also found that the miR-1296 high expressing HCC tissues showed increased expression of E-cadherin and decreased expression of Vimentin. These results suggest that miR-1296 inhibits HCC metastasis by suppressing EMT phenotype.

SRPK1, a highly conserved protein in precursor mRNA translation and splicing, chromatin reconstruction, is dysregulated in different cancers [26, 35]. Moreover, SRPK1 play a critical role in EMT process of human glioblastoma [36]. Here, we confirmed that SRPK1 was a direct downstream target of miR-1296 and mediated the biological function of miR-1296 in HCC. First, miR-1296 negatively regulated SRPK1 abundance in HCC cells. Second, the complementary sequences of miR-1296 were identified in the 3’UTR of SRPK1 mRNA. MiR-1296 overexpression or knockdown accordingly altered the luciferase activity of wt 3’UTR but not mt 3’UTR of SRPK1. Third, miR-1296 was inversely correlated with the expressions of SRPK1 in HCC tissues. Next, we demonstrated that SRPK1 mediated miR-1296-modulated migration, invasion and EMT process of HCC cells. Previous study shows that SRPK1 functions as an oncogene via promoting activation of PI3K/AKT signaling [26]. Activation of PI3K/AKT signaling pathway is involved in the development and progression of HCC and regulates the malignant biological function of cancer cells [37, 38]. Moreover, PI3K/AKT plays a crucial role in EMT process of HCC [39]. Herein, we discovered that miR-1296 restrained the activation of PI3K/AKT signaling. The activator of PI3K/AKT pathway abrogated the inhibitory effect of miR-1296, while AKT inhibitor reversed the promoting effects of miR-1296 knockdown on migration, invasion and EMT process of HCC cells. Therefore, SRPK1/AKT pathway may be involved in the role of miR-1296 in HCC cells.

It’s necessary to confirm whether miR-1296 and SRPK1 could serve as valuable biomarkers for diagnosis and prognostic prediction. Here, we found that both low expression of miR-1296 and high level of SRPK1 were significantly associated with adverse clinical features of HCC patients. In addition, we confirmed that miR-1296 underexpression and SRPK1 overexpression as well as their combination were obviously correlated with poor prognosis of HCC patients. These results suggest that miR-1296 and SRPK1 may be promising predictors for the prognosis of HCC patients.

Previous study confirms that serine-arginine (SR) protein phosphorylation is increased in hypoxia condition [28]. SRPK1 dissimilarly impacts the growth, metastasis, chemosensitivity and angiogenesis of glioma in hypoxic conditions [29]. Moreover, hypoxia environment is a critical cause for HCC metastasis and leads to abnormal expression of miRNAs [40, 41]. Therefore, we tried to explore the relationship between hypoxia and miR-1296 in HCC. Our data showed that miR-1296 expression was significantly decreased in hypoxia. Moreover, miR-1296 restoration abolished the promoting effects of hypoxia on migration, invasion and EMT process of HCC cells. These results suggest that hypoxia-induced miR-1296 loss promotes the metastasis and EMT of HCC.

In conclusion, we demonstrate for the first time that miR-1296 is underexpressed in HCC tissues and cell lines, and its reduced expression is correlated with malignant clinicopathological features. Furthermore, we confirm that miR-1296 inhibits migration, invasion and EMT process of HCC cells probably by directly targeting SRPK1-mediated PI3K/AKT pathway. Notably, miR-1296 underexpression, SRPK1 overexpression and their combination are potential prognostic predictors for the survival of HCC patients. Moreover, hypoxia is a key cause for miR-1296 underexpression in HCC cells. In summary, the deregulation of miR-1296 may play an important role in tumor metastasis and may be a novel prognostic factor and potential therapeutic target for HCC.

## Conclusions

To conclude, we recognize miR-1296 underexpression as a biomarker for predicting poor prognosis of HCC patients. The hypoxia-induced miR-1296 loss creates a milieu of metastasis facilitation that plays a promoting role in HCC progression. A mechanism by which miR-1296 inhibits EMT and metastasis probably by targeting SRPK1-mediated PI3K/AKT pathway plays an important role in this process. This finding will improve understanding of mechanism involved in cancer progression and provide novel targets for the molecular treatment of HCC.

## Additional files

Additional file 1: Figure S1. miR-1296 suppresses EMT process of HCC cells. (A) HCCLM3 cells that were transfected with miR-1296 and miR-control, respectively, were subjected to immunoblotting for the expression of EMT-related markers including ZO-1, ZEB-1, Slug, Snail and Twist. (B) miR-1296 knockdown decreased ZO-1 expression and increased the levels of ZEB-1, Slug, Snail and Twist in Hep3B cells. *P < 0.05. (TIFF 258 kb)

Additional file 2: Figure S2. IHC staining of E-cadherin, Vimentin and SRPK1 in HCC tissues. Representative IHC results indicated that strong staining of E-cadherin and weak staining of Vimentin and SRPK1 were observed in miR-1296 high-expressing HCC tissue. Weak staining of E-cadherin and strong staining of Vimentin and SRPK1 were presented in miR-1296 low-expressing HCC tissues. (TIFF 2252 kb)

Additional file 3: Figure S3. miR-1296 does not regulate the expression of other predicted targets in HCC cells. (A) HCCLM3 cells that were transfected with miR-1296 and miR-control, respectively, were subjected to immunoblotting for the expression of ERBB2, CCND1 and MCM2. (B) miR-1296 knockdown didn’t obviously increased the levels of ERBB2, CCND1 and MCM2 protein in Hep3B cells. (TIFF 242 kb)

Additional file 4: Figure S4. SRPK1 faciliates migration, invasion and EMT progression of HCC cells. (A) HCCLM3 cells that were transfected with SRPK1 siRNA or control siRNA were detected by immunoblotting. (B) SRPK1 silencing notably restrained migration and invasion of HCCLM3 cells. (C) SRPK1 was overexpressed by plasmid transfection and confirmed by western blotting in Hep3B cells. (D) SRPK1 restoration enhanced the migratory and invasive abilities of Hep3B cells. (E) SRPK1 knockdown led to increase of E-cadherin expression and decrease of N-cadherin and Vimentin in HCCLM3 cells. (F) SRPK1 overexpression promoted the EMT progression of Hep3B cells. *P < 0.05. (TIFF 3508 kb)

Additional file 5: Figure S5. The prognostic significance of miR-1296 and SRPK1 in another cohort of HCC patients. (A) and (B) OS and DFS were compared between miR-1296 high expressing HCC patients and low expressing cases. (C) and (D) OS and DFS were compared between SRPK1 high expressing HCC patients and low expressing cases. (E) and (F) OS and DFS were compared between four subgroups of HCC patients (subgroup I: high miR-1296/low SRPK1; subgroup II: low miR-1296 /low SRPK1; subgroup III: high miR-1296/high SRPK1; subgroup IV: low miR-1296/high SRPK1). For each cohort, subgroups were divided according to the cutoff values, which were determined as the median level of miR-1296 and SRPK1 in HCC tissues. **P < 0.01. (TIFF 213 kb)

Additional file 6: Figure S6. SRPK1/AKT axis functions in hypoxia-induced metastasis and EMT process of HCC cells. (A) The levels of SRPK1 and p-AKT were increased after exposing to hypoxia condition in Hep3B cells. (B) and (C) Hypoxia induced migration and invasion of Hep3B cells. While, either SRPK1 knockdown or MK2206 treatment blocked the pro-metastatic effects of hypoxia in Hep3B cells. (D) Hypoxia induced EMT progression of Hep3B cells. Whereas, either SRPK1 knockdown or MK2206 treatment prohibited the EMT progression of Hep3B cells under hypoxia condition. *P < 0.05. (TIFF 5115 kb)

## Abbreviations

3′-UTR: 3′-untranslated region
EMT: Epithelial–mesenchymal transition
H&E: Hematoxylin and eosin
HCC: Hepatocellular carcinoma
IF: Immunofluorescence
IHC: Immunohistochemistry
miRNAs: MicroRNAs
qRT-PCR: Real-time quantitative reverse transcription polymerase chain reaction
SRPK1: Serine-arginine protein kinase 1
TNM: Tumor-node-metastasis
