# Supplementary figures and images for: MicroRNA-1296 inhibits metastasis and epithelial-mesenchymal transition of hepatocellular carcinoma by targeting SRPK1-mediated PI3K/AKT pathway
Source: Mol Cancer. 2017 Jun 12;16:103. doi: 10.1186/s12943-017-0675-y (PMC5469159; doi:10.1186/s12943-017-0675-y)

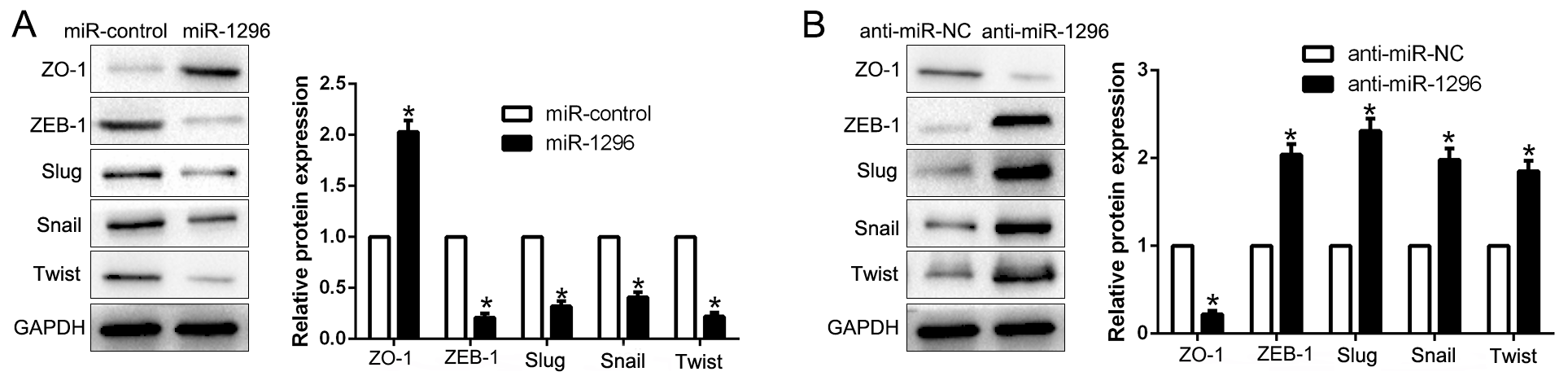

Supplement: Supplementary file 1 — miR-1296 suppresses EMT process of HCC cells. (A) HCCLM3 cells that were transfected with miR-1296 and miR-control, respectively, were subjected to immunoblotting for the expression of EMT-related markers including ZO-1, ZEB-1, Slug, Snail and Twist. (B) miR-1296 knockdown decreased ZO-1 expression and increased the levels of ZEB-1, Slug, Snail and Twist in Hep3B cells. *P < 0.05. (TIFF 258 kb) [file 12943_2017_675_MOESM1_ESM.tif]

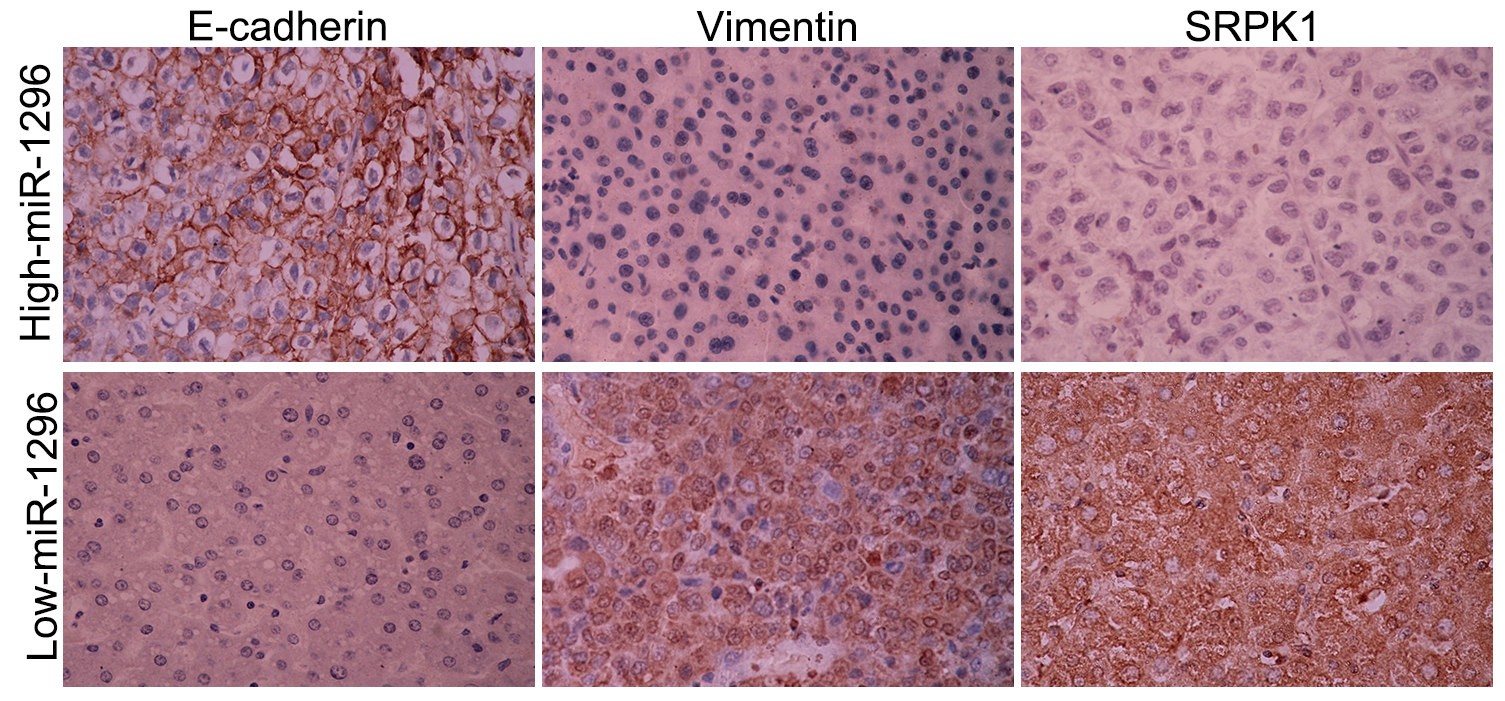

Supplement: Supplementary file 2 — IHC staining of E-cadherin, Vimentin and SRPK1 in HCC tissues. Representative IHC results indicated that strong staining of E-cadherin and weak staining of Vimentin and SRPK1 were observed in miR-1296 high-expressing HCC tissue. Weak staining of E-cadherin and strong staining of Vimentin and SRPK1 were presented in miR-1296 low-expressing HCC tissues. (TIFF 2252 kb) [file 12943_2017_675_MOESM2_ESM.tif]

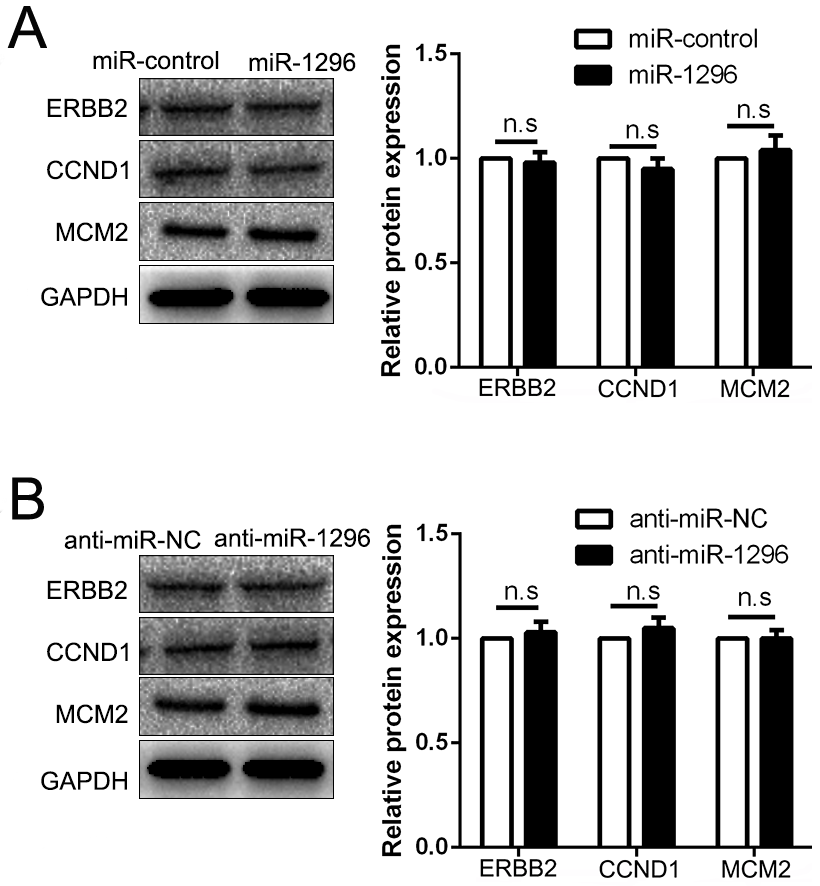

Supplement: Supplementary file 3 — miR-1296 does not regulate the expression of other predicted targets in HCC cells. (A) HCCLM3 cells that were transfected with miR-1296 and miR-control, respectively, were subjected to immunoblotting for the expression of ERBB2, CCND1 and MCM2. (B) miR-1296 knockdown didn’t obviously increased the levels of ERBB2, CCND1 and MCM2 protein in Hep3B cells. (TIFF 242 kb) [file 12943_2017_675_MOESM3_ESM.tif]

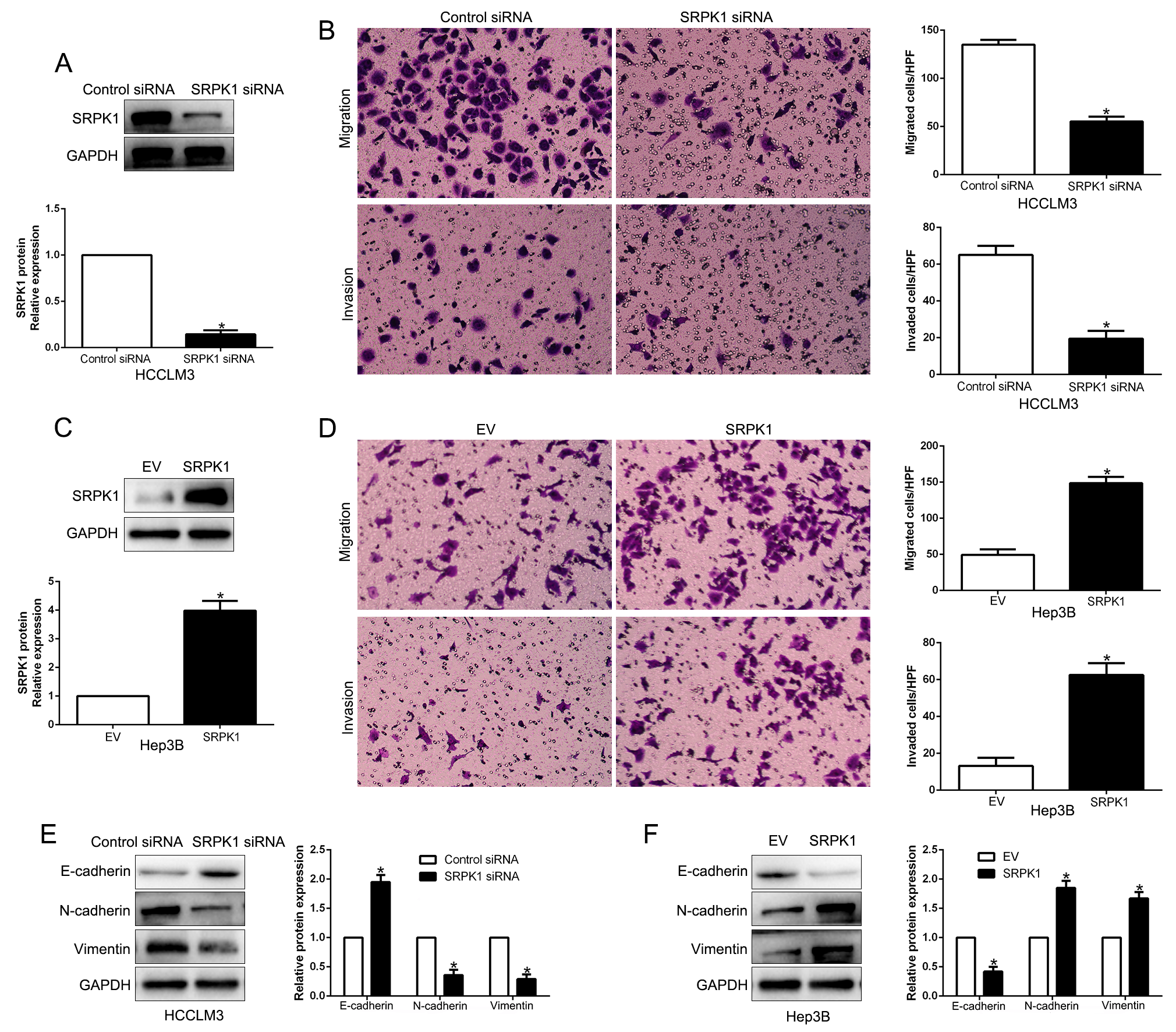

Supplement: Supplementary file 4 — SRPK1 faciliates migration, invasion and EMT progression of HCC cells. (A) HCCLM3 cells that were transfected with SRPK1 siRNA or control siRNA were detected by immunoblotting. (B) SRPK1 silencing notably restrained migration and invasion of HCCLM3 cells. (C) SRPK1 was overexpressed by plasmid transfection and confirmed by western blotting in Hep3B cells. (D) SRPK1 restoration enhanced the migratory and invasive abilities of Hep3B cells. (E) SRPK1 knockdown led to increase of E-cadherin expression and decrease of N-cadherin and Vimentin in HCCLM3 cells. (F) SRPK1 overexpression promoted the EMT progression of Hep3B cells. *P < 0.05. (TIFF 3508 kb) [file 12943_2017_675_MOESM4_ESM.tif]

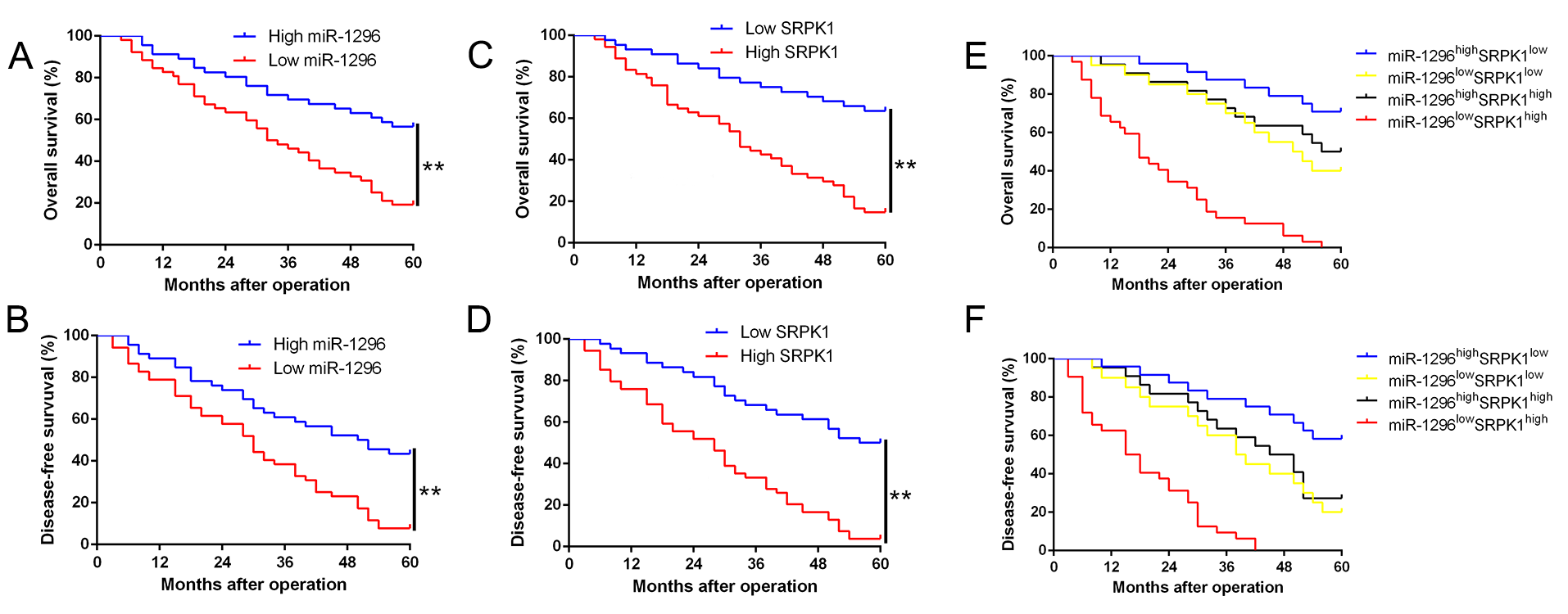

Supplement: Supplementary file 5 — The prognostic significance of miR-1296 and SRPK1 in another cohort of HCC patients. (A) and (B) OS and DFS were compared between miR-1296 high expressing HCC patients and low expressing cases. (C) and (D) OS and DFS were compared between SRPK1 high expressing HCC patients and low expressing cases. (E) and (F) OS and DFS were compared between four subgroups of HCC patients (subgroup I: high miR-1296/low SRPK1; subgroup II: low miR-1296 /low SRPK1; subgroup III: high miR-1296/high SRPK1; subgroup IV: low miR-1296/high SRPK1). For each cohort, subgroups were divided according to the cutoff values, which were determined as the median level of miR-1296 and SRPK1 in HCC tissues. **P < 0.01. (TIFF 213 kb) [file 12943_2017_675_MOESM5_ESM.tif]

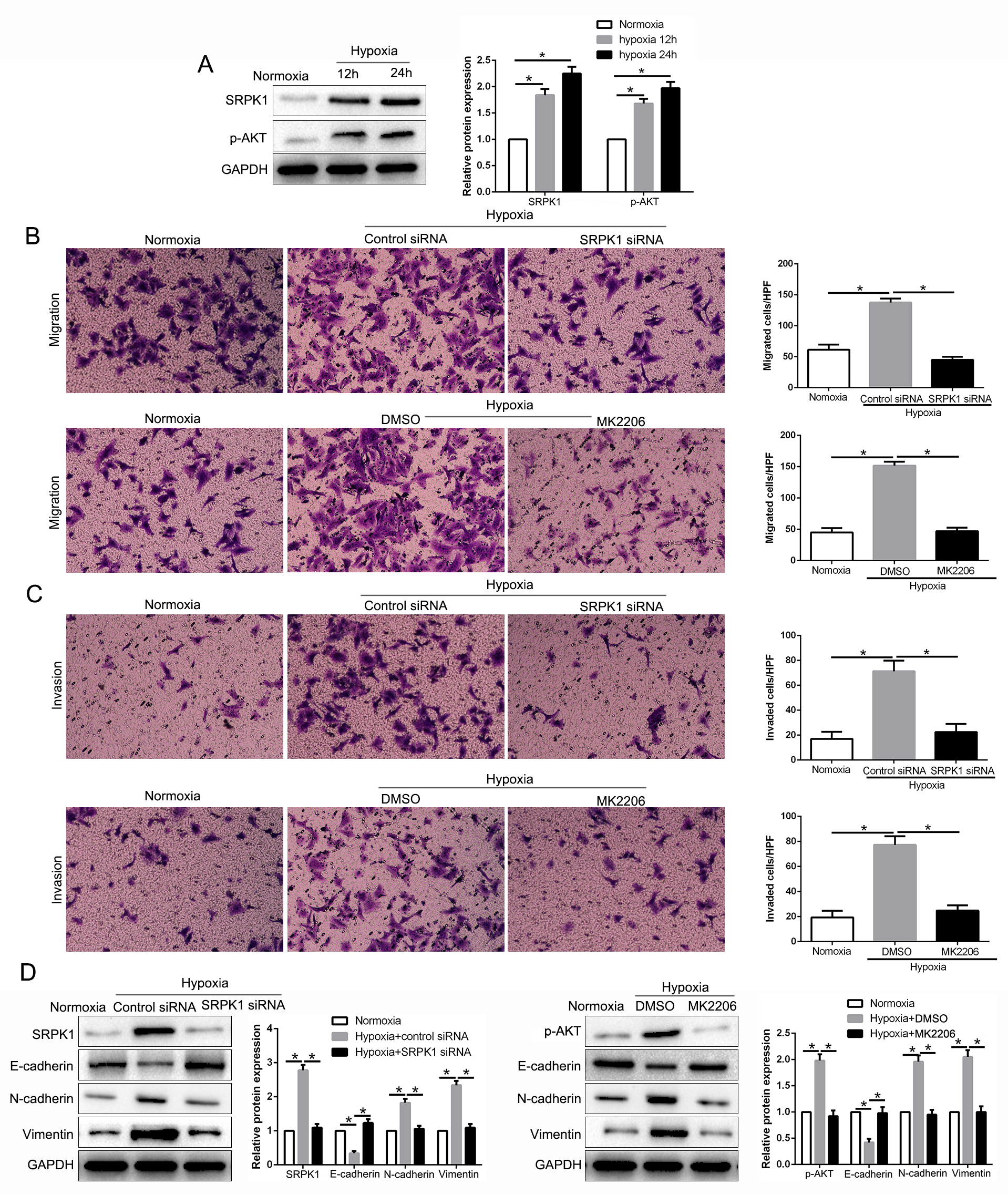

Supplement: Supplementary file 6 — SRPK1/AKT axis functions in hypoxia-induced metastasis and EMT process of HCC cells. (A) The levels of SRPK1 and p-AKT were increased after exposing to hypoxia condition in Hep3B cells. (B) and (C) Hypoxia induced migration and invasion of Hep3B cells. While, either SRPK1 knockdown or MK2206 treatment blocked the pro-metastatic effects of hypoxia in Hep3B cells. (D) Hypoxia induced EMT progression of Hep3B cells. Whereas, either SRPK1 knockdown or MK2206 treatment prohibited the EMT progression of Hep3B cells under hypoxia condition. *P < 0.05. (TIFF 5115 kb) [file 12943_2017_675_MOESM6_ESM.tif]
